# PDTR: Probabilistic and Deterministic Tree-based Routing for Wireless Sensor Networks

**DOI:** 10.3390/s20061697

**Published:** 2020-03-18

**Authors:** Rafia Ghoul, Jing He, Sana Djaidja, Mohammed A. A. Al-qaness, Sunghwan Kim

**Affiliations:** 1 College of Computer Science and Electronic Engineering, Hunan University, Changsha 410006, China; Tigre.eco@live.fr (R.G.); jhe@hnu.edu.cn (J.H.); 2 Faculty of medicine Mohamed Maherzi Ex-Laperine, University of Algiers 1, Algiers 16000, Algeria; sanadja1992@gmail.com; 3 State Key Laboratory for Information Engineering in Surveying, Mapping and Remote Sensing, Wuhan University, Wuhan 430079, China; alqaness@whu.edu.cn; 4 School of Electrical Engineering, University of Ulsan, Ulsan 44610, Korea

**Keywords:** wireless sensor networks, routing, probabilistic routing table, hops-count, energy efficiency, network lifetime, transmission distance, residual energy, probability distribution

## Abstract

This paper proposed a “Probabilistic and Deterministic Tree-based Routing for WSNs (PDTR)”. The PDTR builds a tree from the leaves to the head (sink), according to the best elements in the initial probabilistic routing table, measured by the product of hops-count distribution, and transmission distance distribution, to select the best tree-paths. Each sender node forwards the received data to the next hop via the deterministic built tree. After that, when any node loses of its energy, PDTR updates the tree at that node. This update links probabilistically one of that node’s children to a new parent, according to the updated probabilistic routing table, measured by the product of the updated: Hops-count distribution, transmission distance distribution, and residual energy distribution at the loss of $\ell_{e}$ energy. By implementing the control parameters in each distribution, PDTR shows the impact of each distribution in the routing path. These control parameters are oriented by the user for different performances. The simulation results prove that selecting the initial best paths to root the packets via unicast, then improving the tree at the node with loss of energy by rooting the packets via anycast, leads to better performance in terms of energy consumption and network lifetime.

## 1. Introduction

A wireless sensor network (WSN) is a network of low-power and low-cost devices that communicate among themselves within the communication range using radio signals. Many protocols have been proposed to maximize both energy efficiency and energy balancing. WSNs have generated an increasing interest because of their significant role in our daily lives: i.e., surveillance, health care, acoustic and seismic detection, environmental monitoring. Most of these protocols are location-based schemes, characterized by three facts: first, each node in the network must be aware of its location information. Second, each node must be aware of its direct neighbor node’s location. Third, the source node must be aware of the destination node’s location [1]. The localization of WSNs is a very crucial issue; many methods have been proposed to get the node’s location, and most of these proposed methods supposed that only a small proportion of sensor nodes, called anchor (beacon) nodes, are location-aware through a global positioning system (GPS), because of the high cost and energy consumption of GPS. However, the other nodes could obtain their position by estimating distance, using the anchor nodes positions, through other localization technologies [2,3]. Other proposed WSN localization technologies are based on different measurement techniques such as the time-of-arrival (TOA), angle-of-arrival (AOA), and received signal strength indicator (RSSI) [4,5,6,7]. Later, in [8,9], the cooperative localization technology was developed for hybrid networks containing mobile and static sensors. Recently, in [10], the authors proposed a centralized expectation maximization-based passive localization technology for asynchronous receivers (EMplLaR). Being aware of the nodes’ location allows the use of many power control techniques to reduce energy consumption.

Data forwarding toward the sink via deterministic protocols may cause quick energy depletion for some nodes, due to the sender node’s forwarder unique choice, which causes a topology disruption, thus a quick network death. Meanwhile, the probabilistic routing approach came with the improvement, such that each sensor has to select its forwarder from a set of its candidates according to their routing probabilities, based on its neighborhood knowledge, thus providing a longer network lifetime. 

From the existing probabilistic and deterministic protocols, we concluded that to minimize the energy consumption and prolong the network lifetime, the protocol should depend on three factors. Reference [11] proposed a Hop-based Routing Protocol based on an Energy Efficient Minimum Spanning Tree for WSN. From the simulation results, it was proved that the proposed protocol improved the network in terms of energy consumption and lifetime extension, hence the importance of the hop-count factor. Article [12] also considered this factor in the presented protocol called (STDD). The authors in [13] showed that the Euclidian distance has a direct effect on energy consumption—the more the distance is minimized, the more the consumed energy is reduced. In [5,6], a p-persistence protocol was proposed, such that the forwarding probability is calculated according to the distance between two nodes. Thus, it uses the relative distance between two nodes for retransmission delay adjustment. Thus, the importance of the transmission distance factor is shown.

In WSNs, ensuring energy efficiency does not mean ensuring energy balancing. Hence, to balance the energy dissipation, the selection of the routing protocol should be performed to save the residual energy in each node, as much as possible. Article [14] proposed a Distance-based Energy-Aware Routing (DEAR) algorithm. It ensures energy efficiency, energy balancing, and takes into consideration two factors: distance and residual energy. DEAR simulation provided better results in terms of energy balancing and consumption. Reference [15] proposed a tree-based protocol that considers both the number of intermediate nodes to the sink, and the residual energy for each node. The proposed protocol increases the network lifetime comparatively than the previously designed protocols; this may be explained by the fact that it reduces the energy of the excessive messages between nodes. Hence, the importance of the last factor, the residual energy, for the lifetime extension is demonstrated here. The authors in [11,16,17] also considered this factor in their presented protocols. 

The main purpose of tree-based protocols is to ensure that no loops will be created when we have redundant paths in the network; with tree-based protocols, it is possible to avoid packet collisions. In [18], the authors proposed a new tree-based routing algorithm that reduces energy consumption. The presented algorithm is a new cluster-based that uses a tree structure for packet transmission. It was designed in the manner to first minimize the used hop-lengths for data transmission inside the clusters, and then select the nearest cluster head to the base station. Reference [19] proposed PLCTA, a probabilistic load-balancing converge-cast tree, which considers the impact of sensors heterogeneities. It uses a weight assignment method, and for each link (node-node’s candidate parent) there is a weight assignment, and over time this weight is adjusted according to local information. From the simulation, PLCTA achieved better performance in both energy efficiency and lifetime. In tree-based protocols, each time the node has to send the received data to a fixed next hop, which may cause quick energy depletion for some nodes. In consequence, updating the tree is required in order to save their batteries [15].

In this work, we propose a Probabilistic and Deterministic Tree-based Routing protocol (PDTR), which considers both the optimal routing path and energy balance between nodes. It consists of three different stages: tree construction, data transmission, and residual energy saving. PDTR starts with the selection of the optimal paths to root the packets via unicast, in order to optimize energy consumption. Rooting the packets via a deterministic tree reduces the energy consumption in comparison to the probabilistic tree. However, it may cause a quick depletion of the energy for some nodes. To fix this problem, PDTR is proposed to save the energy of any node which loses of its energy, in order to balance the energy in the network. This strategy will probabilistically root the packets, from this node to the forwarder (anycast). The protocol with the two strategies, the deterministic and probabilistic tree, has provided better results when compared with other protocols: DHA, DRP, and DAPR. 

The rest of this paper is organized as follows. Section 2 introduces some related work of the WSN designed routing protocols. Section 3 presents the used Radio Model. In Section 4, a description of the proposed PDTR is presented. Performance evaluation and comparison are given in Section 5 and Section 6, respectively, while Section 7 concludes this paper.

## 2. Related Work 

Routing protocols for WSNs are divided into three main categories: network organization-based routing protocols, where the protocols are classified as flat-based routing, hierarchical-based routing, and location-based routing (geo-centric); route discovery-based routing protocols, where the protocols are classified as reactive protocols, proactive protocols, and hybrid protocols; and operation-based routing protocols, where the protocols are classified as multi-path routing protocols, query-based routing protocols, negotiation-based routing protocols, QoS-based routing protocols, and coherent data processing routing protocols [20]. Designing a successful protocol for WSNs must achieve both energy efficiency and balance. A wide range of researchers have focused on energy efficiency, but few of them have focused on energy balancing (network lifetime). The authors in [21] proposed the Enhanced tree routing protocol (ETR), which improves the traditional Tree Routing protocol (TR), by the fact that ETR paths are built according to the parent-child links, and other one-hop neighbors links, to reach shorter paths. ETR simulation showed more energy efficiency, comparatively, than TR simulation; this is explained by the fact of using the address structure to select ETR paths. The article [14] proposed Distance-based Energy-Aware Routing (DEAR). From the simulation results, it is shown that DEAR reduces and balances the energy consumption in comparison to the previously proposed protocols. The achieved results are explained by the fact that DEAR takes into consideration, first, the distance distribution, and second, the residual energy.

One year later, a proposed Energy-Balanced Routing Protocol (EBRP) was proposed in [22]. EBRP simulation results show better performance in term of energy balancing. This is explained by the fact that this proposed protocol takes into consideration the depth, the energy density, and the residual energy. Later, the authors in [23] introduced a probabilistic tree-based routing protocol (TBRR), that processes in two stages. The packets in TBRR protocol are rooted toward the sink under a highly reliable manner based on the node degree and link loss. From the simulation, the TBRR protocol improved the calculation of forwarding probability. Two years later, in [24], a designed Self-Organized Tree-Based Energy-Balance routing protocol (GSTEB) was presented. In the GSTEB protocol, the base station selects the root for nodes, and each node is aware of itself and its neighbors’ information. The simulation results demonstrate a better performance in term of energy balancing. This can be explained by the fact that GSTEB is a dynamic protocol. In [25], the authors presented a quadrant-based routing protocol that divides the network area into four quadrants. The selection of the parent node is based on the distance, and residual energy simulation results prove better performance in terms of energy efficiency, with respect to load balancing.

Recently, the authors in [26] developed an Energy Efficient Balanced Tree-Based Routing Protocol for Wireless Sensor Network (EEBTR) that structures a balanced-tree scheme by proceeding in two phases—the Up-Down Balancing phase (UDB) and Down-Up Balancing phase (DUB)—to balance between number of hops and energy loading in the network. The EEBTR results show better performance in terms of energy balancing and network lifetime. 

Finally, Routing protocols in WSNs are classified into two main approaches: the deterministic protocol approach, and probabilistic protocol approach [27]. The protocols cited above are deterministic protocol approaches. In the deterministic approach, the forwarder node for each node is already fixed, and the routing paths are predefined. This may cause quick energy depletion, which can result in the loss of some nodes and a loss of part of the network.

In [28], the authors proposed a probabilistic scheme based on the node degree and link loss to increase the reliability of selective forwarding. The authors in [29] presented a Probabilistic Geographic Routing (PGR) that uses only local information, link reliability, and residual energy to probabilistically forward data after selecting a candidate’s nodes and assigning these probabilities to these candidates.

Distributed Heuristic Algorithm (DHA) was presented in [30]. DHA is a probabilistic scheme based on three distances parameters to select the forwarder nodes, the distance between the sender node and the sink, the distance between the forwarder node and the sink, and the maximum distance between the sender node and the sink.

## 3. Preliminaries

### 3.1. Assumptions and Notations

**Figure 1:**
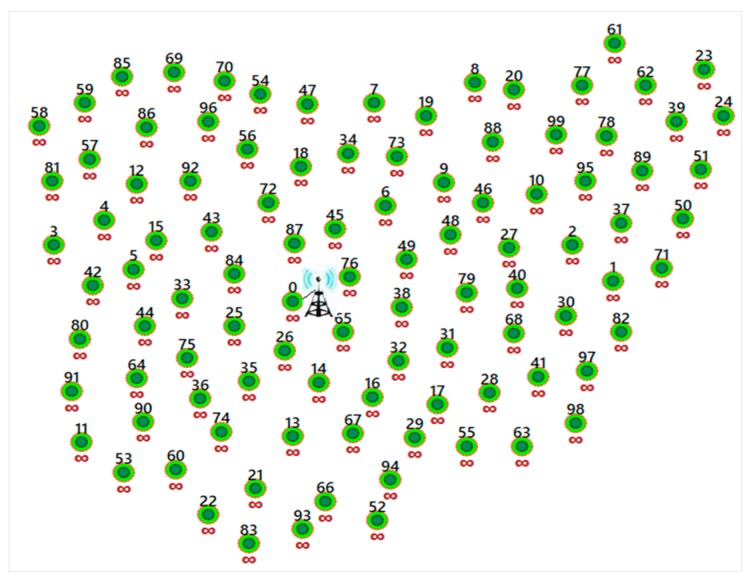
Static wireless sensor network (WSN) with 99 homogeneous sensors and the centralized sink node (node 0).

This proposed protocol, PDTR, was designed under the following conditions; the non-mobility of the sink and the sensors, and the homogeneity of all the network sensors (the same initial energy and the same communication range), except for the sink, which is charged with high energy, (see Figure 1).

Figure 1 shows the PDTR working network. The sink (node 0) is static and located at the center, near to the base station. The sensors are homogeneous and static. The sensors near the sink (within the communication range) communicate directly with the sink. The sensors out of the sink communication range should maintain a multi-hop path for the communication with the sink. The main objective of PDTR is to prolong the network lifetime under the conditions depicted and explained in Figure 1. Table 1 shows the notations that used in this study. 

### 3.2. Radio Model Used for PDTR

**Figure 2:**
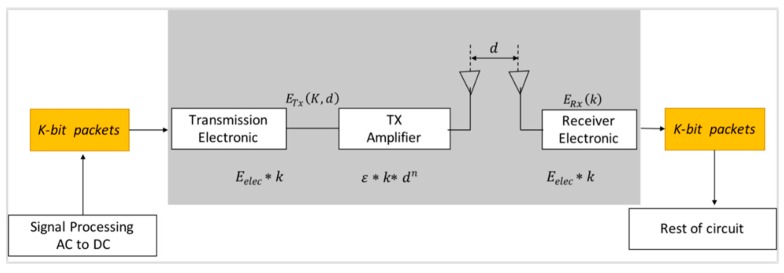
Radio Model used for Probabilistic and Deterministic Tree-based Routing for WSNs (PDTR).

The proposed PDTR uses First Order Radio Model with asymmetric channel to run the radio as depicted in Figure 2, such that it uses the same energy, to transmit the data from x to y, and to transmit the same data from y to x, this energy is denoted by *E_elec_*, and has the value of *E_elec_* = $50\frac{nJ}{bit}$. *E_elec_* depends on filtering, modulation, digital coding and signal spreading [26]. In this model, two channel models are used. The Free space channel model of transmitter amplifier is denoted by *ε_fs_*, and has the value of *ε_fs_* = 10 *pJ/bit*/*m*^2^. The Multi-path channel model of transmitter amplifier is denoted by $\varepsilon_{mp}$ and has the value $\varepsilon_{mp}=0.0013\;pJ/bit/m^{4}$ [26]. Let $d_{\left(n_{i},n_{j}\right)}$ be the distance between the transmitter $n_{i}$ and the receiver $n_{j}$. Let $t_{0}\;$be the distance threshold, and it has the value of $t_{0}=\sqrt{\frac{\varepsilon_{fs}}{\varepsilon_{mp}}}$ (meters).

When $d_{\left(n_{i},n_{j}\right)}$ is greater than $t_{0}=\sqrt{\frac{\varepsilon_{fs}}{\varepsilon_{mp}}}$ (meters), a multi-path (mp) channel model is used to run the radio. Otherwise, the free space (fs) channel model is used to run it, as given in Equations (1) and (2). Each sensor needs an energy of $E_{Tx}\left(\langle n_{i},n_{j}\rangle ,k\right)$ to transmit k bits size message over distance$\;d_{\left(n_{i},n_{j}\right)}$, and needs an energy of $E_{\mathrm{Rx}}\left(k\right)$ to receive the transmitted message of k bits.

(1) $$E_{Tx}\left(n_{i},n_{j},k\right)=\{\begin{array}{c} \left(k\cdot E_{elec}\right)+\left(k\cdot \varepsilon_{fs}\cdot {\left[d_{\left(n_{i},n_{j}\right)}\right]}^{2}\right),d_{\left(n_{i},n_{j}\right)}<t_{0}\\ \left(k\cdot E_{elec}\right)+\left(k\cdot \varepsilon_{mp}\cdot {\left[d_{\left(n_{i},n_{j}\right)}\right]}^{4}\right),d_{\left(n_{i},n_{j}\right)}\geq t_{0} \end{array}$$

(2) $$\;E_{Rx}\left(n_{j},k\right)=k\cdot E_{elec}$$

## 4. Proposed Method PDTR

This work is categorized among the location-based deterministic and probabilistic protocols. It proposes a probabilistic and deterministic tree-based routing approach called PDTR, which aims to attend an efficient and balanced energy consumption. It is built from the leaves to the root, the sensing nodes (leaves) transmit the sensed data to the sink (root) node, through a deterministic tree structure. Each node selects a parent with a higher probability in the initial probabilistic routing table, to relay the sensed data toward the sink. After time, when any node $n_{i}$ loses $\ell_{e}\;$of its energy, the sub-tree routed at the node $n_{i}\;$cuts one of its sub-sub-trees, and the root node of the cut sub-subtree selects probabilistically a new parent, according to the updated probabilistic routing table. Then, the sub-subtree routed at a cut node will be rooted probabilistically at the new parent node. This process is repeated for the node $n_{i},\;$each time it loses $\ell_{e}\;$of its energy, starting from the first energy loss, until the value of its remaining energy is less than $\ell_{e}$. The used probabilistic protocol in the removed child is classified among the adaptive probabilistic schemes. Each node $n_{i}$ is aware of its location $\left(x_{i},y_{i}\right)$, and has a hop-count $h\left(n_{i}\right)$ that indicates the minimum number of hops required to reach the sink node, as defined in Table 1. All sensors in the network are fully charged with the same initial energy$\;E^{*}$, except for the sink node, which has a different initial energy, $E_{n_{0}}{}^{*}$, as given in Table 2. Every source node collects data and has to choose the appropriate forwarder to send the collected data to, according to the probabilistic routing table.

### 4.1. The Probabilistic Routing Table Set-Up Phase

This phase is achieved according to three selection distribution: the hop-count distribution, the sender node chooses the neighbor with lower hop-count to relay the sensed data. In the transmission distance distribution, the sender node selects the nearest neighbor to be the next hop (forwarder). Finally, in the residual energy distribution, the node with the greater residual energy has more chance to be chosen as the next-hop, if its energy has not fallen below threshold energy.

#### 4.1.1. Hop-count Distribution

The source node selects the neighbor node with a lower hop-count as its forwarder to relay the sensed data. This distribution has the aim to give priority to nodes close to the sink; the closer the forwarder node is to the sink, the less energy is needed for data transmission, and the priority is higher for the forwarders that are closer to the sink. We define $h\left(n_{i}\right)\;$by the hop-count of the node $n_{i}$ as the minimum number of hops to reach the sink. In ℕ sensor nodes network, the maximum hop-count can be ℕ-1, because at the worst case, each node has only two neighbor nodes, a back-warder node and forwarder node, except for the sink node, which does not have a forwarder (parent), and source nodes do not have a back-warder. Here, the hop-count of the node $n_{i}$ neighboring nodes is defined as a random variable: $h\left(n_{l}\right)=\left(h\left(n_{1}\right),\ldots ,h\left(n_{m_{i}}\right)\right),m_{i}\;neighbors$. 

Let ${\mathbb{N}}_{i}\;$be the neighboring nodes set of $n_{i}\;$such that$\left|{\mathbb{N}}_{i}\right|=m_{i},$ as defined in Table 1.

The hop-count function is normalized by the following Equation (3).

(3) $$\bar{h}\left(n_{j}\right)=1+\left(\frac{h\left(n_{j}\right)}{\mathbb{N}-1}\right)\;\forall n_{j}\in {\mathbb{N}}_{i}$$

The forwarding probability function of the hop-count distribution from the sender $n_{i}$ to its neighboring nodes is calculated by Equation (3) and given by the following Equation (4).

(4) $$\overset{ˇ}{h}\left(n_{i,j}\right)=\frac{\bar{h}\left(n_{j}\right)}{\sum_{l=1}^{m_{i}}\bar{h}\left(n_{l}\right)},\;\forall n_{j}\in {\mathbb{N}}_{i},\;\left|{\mathbb{N}}_{i}\right|=m_{i\;}$$

From Equation (4), we can see that further nodes have bigger probabilities, but we want to give a higher probability to the closer nodes. Let $\tilde{h}\left(n_{j}\right)$ be the mass probability of the hop-count distribution, such that the higher probability is given to the nodes with lower hop-count, as expressed in Equation (5).

(5) $$\tilde{h}\left(n_{i,j}\right)=\frac{{\left(1-\overset{ˇ}{h}\left(n_{i,j}\right)\right)}^{\alpha }}{\sum_{l=1}^{m_{i}}{\left(1-\overset{ˇ}{h}\left(n_{i,l}\right)\right)}^{\alpha }},\;\forall n_{j}\in {\mathbb{N}}_{i}$$

To determine the impact of the hop-count distribution on the other two distributions defined in the proposed PDTR, we defined in Table 1, a hop-count control parameter denoted by *α* such that: *α* ≥ 0, as shown in Equation (5).

#### 4.1.2. Transmission Distance Distribution

The closer the forwarder node is to the sender; the less energy is needed for data transmission. Higher priority is given to forwarders closer to the sender node. From each node to its neighboring nodes, the transmission distance can be calculated by the Euclidian distance, and it is normalized by Equation (6). The furthest node can have a maximum distance of communication radius δ value from the sender node.

Let ${\mathbb{N}}_{i}$ be the neighboring nodes of the node $n_{i}$, such that $\left|{\mathbb{N}}_{i}\right|=m_{i};d_{i,l}=\left(d_{i,1},d_{i,2},\ldots ,d_{i,m_{i}}\right),m_{i}\;neighboring\;node.$ The transmission distance distribution function is normalized by Equation (6).

(6) $${\bar{d}}_{i,j}=1+\left(\frac{d_{i,j}}{\delta }\right)\;\forall n_{j}\in {\mathbb{N}}_{i}$$

The probability function of the transmission distance distribution by using Equation (6) is given by Equation (7).

(7) $${\overset{ˇ}{d}}_{i,j}=\frac{{\bar{d}}_{i,j}}{\sum_{l=1}^{m_{i}}{\bar{d}}_{i,l}},\forall n_{j}\in {\mathbb{N}}_{i}$$

From Equation (7), we can see that the further nodes have higher probabilities, but we want to give a higher probability to the closer nodes.

Let ${\tilde{d}}_{i,j}$ be the mass probability function of the distance distribution (see Equation (8)), such that the higher probability will be given to the closer nodes to the sender node, the smaller distance will have a greater probability. To determine the impact of the transmission distribution on the other two distributions defined in the proposed PDTR, we defined in Table 1, a transmission distance control parameter denoted by β such that: β≥0, as shown in Equation (8).

(8) $${\tilde{d}}_{i,j}=\frac{{\left(1-{\overset{ˇ}{d}}_{i,j}\right)}^{\beta }}{\sum_{l=1}^{m_{i}}{\left(1-{\overset{ˇ}{d}}_{i,l}\right)}^{\beta }}$$

#### 4.1.3. Residual Energy Distribution

The sender node selects the next-hop with higher remaining residual energy to relay collected data, in order to balance the energy and ensure more lifetime for the network. This distribution has the aim of giving priority to nodes with higher residual energy. However, on the other side, this distribution depends on the location of the nodes, and the nodes with low battery may be used as a relay node. Let $E_{j}$ be the residual energy of the node $n_{j}\;$such that; $n_{j}\;is\;$the forwarder of $n_{i};\;E_{i}=\left(E_{i,1},E_{i,2},\ldots ,E_{i,m_{i}}\right),\;and\;n_{i}\;$has $m_{i}$ neighboring nodes denoted by the set ${\mathbb{N}}_{i}$. Let $\bar{E_{j}}$ be the normalized function of these nodes, given by Equation (9).

(9) $$\bar{E_{j}}=1+\left(\frac{E_{j}}{E^{*}}\right),\forall n_{j}\in {\mathbb{N}}_{i}$$

Let ${\tilde{E}}_{i,j}$ be the mass probability of the residual energy distribution of forwarding data from the sender node to its neighboring nodes, such that the greater probability is given to the nodes with greater residual energy, as expressed in Equation (10).

(10) $${\overset{ˇ}{E}}_{i,j}={\tilde{E}}_{i,j}=\frac{{\bar{E}}_{i,j}{}^{\gamma }}{\sum_{l=1}^{m_{i}}{\bar{E}}_{i,l}{}^{\gamma }},\;\;\;\forall n_{j}\in {\mathbb{N}}_{i}$$

To determine the impact of the residual energy distribution on the other two distributions defined in the proposed PDTR, we defined in Table 1, a residual energy control parameter denoted by *γ* such that: *γ* ≥ 0, as shown in Equation (10).

#### 4.1.4. Initialization of the Probabilistic Routing Table

The probabilistic routing table is a set of elements: ${\tilde{\varphi }}_{i,j},$ for each possible link $n_{i},n_{j},\forall n_{i}\in \mathbb{N},\forall n_{j}\in {\mathbb{N}}_{i},$ such that ${\;\varphi \;˜}_{i,j}$ is calculated by using ${\overset{ˇ}{\varphi }}_{i,j},\;$as explained in Equations (11) and (12).

**Remark** **1.** *The value of*${\tilde{\varphi }}_{i,j}=0$*, if*$n_{j}=n_{i}$*or*$n_{j}\notin {\mathbb{N}}_{i}$;

(11) $${\overset{ˇ}{\varphi }}_{i,j}=\tilde{h}\left(n_{i,j}\right)*{\tilde{d}}_{i,j}*{\tilde{E}}_{i,j}=\;\frac{{\left(1-\overset{ˇ}{h}\left(n_{i,j}\right)\right)}^{\alpha }\;\;{\left(1-{\overset{ˇ}{d}}_{i,j}\right)}^{\beta }\;{\bar{E_{i,j}}}^{\gamma }}{\sum_{l=1}^{m_{i}}{\left(1-\overset{ˇ}{h}\left(n_{i,l}\right)\right)}^{\alpha }\;\sum_{l=1}^{m_{i}}{\left(1-{\overset{ˇ}{d}}_{i,l}\right)}^{\beta }\;\sum_{l=1}^{m_{i}}{\bar{E_{i,l}}}^{\;\gamma }}$$

Hence,

(12) $${\overset{ˇ}{\varphi }}_{i,j}=\frac{{\overset{ˇ}{\varphi }}_{i,j}}{\sum_{n_{j}\in {\mathbb{N}}_{i}}{\overset{ˇ}{\varphi }}_{i,j}}=\;\frac{{\left(1-\overset{ˇ}{h}\left(n_{i,j}\right)\right)}^{\alpha }\;\;{\left(1-{\overset{ˇ}{d}}_{i,j}\right)}^{\beta }\;{\bar{E_{i,j}}}^{\gamma }}{\sum_{l=1}^{m_{i}}{\left(1-\overset{ˇ}{h}\left(n_{i,l}\right)\right)}^{\alpha }\;{\left(1-{\overset{ˇ}{d}}_{i,l}\right)}^{\beta }\;{\bar{E_{i,l}}}^{\;\gamma }}.$$

For initialization, since the initial energy is the same for all nodes in the network (except for the sink node), we can initialize the probabilistic routing table by assuming at the beginning that:

(13) $$\;{\overset{ˇ}{\varphi }}_{i,j}{}^{*}=\tilde{h}{\left(n_{i,j}\right)}^{*}*{\tilde{d}}_{i,j}{}^{*}$$

We note that the hop-count will be defined as depth in a tree structure (since all nodes have the same residual energy distribution). From Equation (13) the initial probabilistic routing table is the set of elements ${\tilde{\varphi }}_{i,j}{}^{*}$, for each possible link $n_{i},n_{j},\;\forall n_{i}\in N,\forall n_{j}\in {\mathbb{N}}_{i}.\;$These elements are calculated by Equation (14).

(14) $${\tilde{\varphi }}_{i,j}{}^{*}=\frac{\;{\overset{ˇ}{\varphi }}_{i,j}{}^{*}}{\sum_{n_{j}\in {\mathbb{N}}_{i}}\;\;{\overset{ˇ}{\varphi }}_{i,j}{}^{*}}=\frac{{\left(1-\overset{ˇ}{h}{\left(n_{i,j}\right)}^{*}\right)}^{\alpha }{\left(1-{\overset{ˇ}{d}}_{i,j}\right)}^{*}{}^{\beta }}{\sum_{l=1}^{m_{i}}\left[{\left(1-\overset{ˇ}{h}{\left(n_{i,l}\right)}^{*}\right)}^{\alpha }{\left(1-{\overset{ˇ}{d}}_{i,l}\right)}^{*}{}^{\beta }\right]}$$

### 4.2. Routing Tree Phase

PDTR builds the tree from the leaves (source node) to the root (sink node). Let $p_{ns}$ be the path starting from the source node $n_{s}$ (located at the head of the path) toward the sink node$\;n_{r}$. Each source node which has sensed data has to send the collected data to one parent that will be selected according to the initial probabilistic routing table; the parent with the highest probability has the priority to be chosen.

**Remark** **2.** *The hops-count notion in the tree is defined as the depth (level). After building the tree, if the initial hops-count for any node is different from its depth (level) in the built tree, the hop-count for that node will be updated to its depth (level) value. Hence, the hops-count distribution has to be updated.*

### 4.3. Updating the Probabilistic Routing Phase

After some time, the residual energy of each node starts to decrease, depending on the quantity of the transmitted packet by each sensor node. Hence, the necessity of updating the tree-routing in order to balance energy consumption and prolong the network lifetime is shown. PDTR proposes a strategy to reach this goal. Each time any node $n_{i}\;$loses $\ell_{e}\;$of its energy, and if it is not a leaf, then it will be defined as a node that needs help, and selects the child with the lowest residual energy to be removed from its children. Then, the selected child node has to probabilistically select a new-parent (forwarder) from its candidate parents, according to the updated probabilistic routing table. This process will be repeated for the node $n_{i}\;\mathrm{each}\;\mathrm{time}\;\mathrm{it}\;$loses $\ell_{e}\;$of its energy, until the value of its remaining energy is less than $\ell_{e}$.

#### 4.3.1. Definitions

**Definition 1.**  *Path Links and Path Nodes: Let* $p_{n_{s}}^{k}$ *be the path transmitting a packet of size k from the source node* $\;n_{s}\;$ *to the sink node* $n_{r}$ *, such that the path links* $p_{n_{s}}^{k}L=\left\{\langle n_{s},n_{v}\rangle ,\langle n_{v},n_{w}\rangle ,\ldots ,\langle n_{l},n_{r}\rangle \right\}$ *are defined as the sequence of all links between each node and its parent, starting from the link* $\langle n_{s},n_{v}\rangle \;$ *between the source node* $n_{s}$ *and its selected forwarder* $n_{v}$ *, to the last link* $\langle n_{l},n_{r}\rangle$ *relaying the last node* $n_{l}$ *to the sink node* $n_{r}$ *. The associated path nodes are defined as* $\;p_{n_{s}}^{k}N$ *, such that:* $p_{n_{s}}^{k}N=\left\{n_{s},n_{v},n_{w},\ldots ,n_{q},n_{f},\ldots ,n_{l},n_{r}\right\}=\left\{n_{s},n_{v},n_{w},\ldots ,n_{q}\right\}⋃\left\{n_{f},\ldots ,n_{r}\right\},$ *such that the node n_f_ is the forwarder node of the node n_q_ and* $p_{n_{s}}^{k}N$ *is expressed in Equation (15).*

(15) $$\;p_{n_{s}}^{k}N=\left\{\bar{n_{s}-n_{q}}\right\}{⋃}^{\;}\left\{\bar{n_{f}-n_{r}}\right\}=p_{n_{s}}^{k}\bar{n_{s}-n_{q}}\;{⋃}^{\;}\;p_{n_{s}}^{k}\bar{n_{f}-n_{r}}$$

**Definition 2.**  *Sub-tree (* $n_{q}$ *) Nodes: Let* $Sbt_{N}^{n_{q}}\;$ *be the Sub-tree (* $n_{q}$ *) Nodes, defined as the nodes of the Sub-tree (* $n_{q}$ *).* $Sbt_{N}^{n_{q}}$ *consists of all paths starting from the source node* $n_{s},\;$ *and passing by the node* $n_{q}$ *toward the sink node* $\;n_{r}$ *, such that* $n_{s}\in S$ *and* S  *is the set of all source nodes sending data, through paths passing by the node* $\;n_{q}\;$ *toward the root (sink) node* $\;n_{r}.\left|S\right|=v$ *, as explained in Equation (16), by using Equation (15).*

(16) $$Sbt_{N}^{n_{q}}=\overset{v}{\underset{s=1}{⋃}}\;p_{n_{s}}^{k}\bar{n_{s}-n_{q}}$$

**Definition 3.**  *Node Need New-Parent Selection: It is clear that the residual energy distribution changes by sending data, which requests an update. Let the node*$n_{h}\;$*be the node that loses*$\ell_{e}$*of its energy, it broadcasts the current energy state to the sink node and to all its children as well. It is declared that*$n_{h}\;$*is the node that needs a help, the neighboring nodes (children nodes) as well, send a feedback to*$n_{h}$*about the current residual energy, then*$n_{h}\;$*selects the child with the lowest residual energy to be removed; the cost of this process is very low and can be negligible. The selected child node is defined as the node needing new-parent, defined as*$n_{np}$.

**Definition 4.**  *Candidate Parents: The selected child node*$n_{np}\;$*broadcasts its current energy state to its neighboring nodes. Then, the neighboring nodes update their residual energy. The residual energy is already updated, once receiving a packet by the node*$n_{np}$*, then*$n_{np}$*selects probabilistically a new-parent node*$n_{p}$*among the set of its candidate parents*$CP_{np}$*based on its updated probabilistic routing table. The*$CP_{np}$*is defined as the set of*$n_{np}\;$*neighboring nodes that do not belong to the Sub-tree (*$n_{h}$*) nodes (*$Sbt_{N}^{n_{h}}$*), and have a higher residual energy than the residual energy of the node*$n_{h}$.

*The candidate parents for* $n_{np}\;\left(CP_{np}\right)\;$ *are a set of neighboring nodes of* $n_{np\;},\;$ *such that they satisfy the conditions (1,2), such that:* $\;\forall \;n_{i\;}\in {\mathbb{N}}_{np},\exists n_{p\;}\in CP_{np}\;$

*1.*  $n_{p}\;\notin \;Sbt_{N}^{n_{h}}\;$ *(The candidate parent should not be a node in the Sub-tree (* $n_{h}$ *) nodes, such that nodes in* $Sbt_{N}^{n_{h}}\;$ *are defined by Equation (16)*
*2.*  $E_{n_{p}}>E_{n_{h}}$*(The residual energy of the candidate parent of the node need new-parent*$n_{p}\;$*should be greater than the residual energy of its current parent (*$n_{h})$).
  *The candidate parents set for*$n_{np\;}$*is denoted by*$CP_{np},\;$*and defined by Equation (17)*.
  (17) $$CP_{np}=\left\{\begin{array}{c} n_{j\;}\backslash n_{j\;}\in {\mathbb{N}}_{np}\&\;n_{j}\notin Sbt_{N}^{n_{nh}}\;\\ \&\;E_{j}>E_{nh}\; \end{array}\right\}$$
*It is clear that* $CP_{np}\subset \;{\mathbb{N}}_{np}.\;$ *Let* $\left|CP_{np}\right|=C_{np}$

**Remark 3**  *If* $\;CP_{np}=\emptyset ,$ $n_{h}\;$ *will select another child node* $n_{\overset{‘}{np}}\;$ *among its children nodes to be removed, such that:* $n_{\overset{‘}{np}}\neq n_{np}\;$ *and* $CP_{\overset{‘}{np}}\neq \emptyset .$

#### 4.3.2. New-Parent Probabilistic Selection

The node needing new-parent $n_{np}$ has to probabilistically select a new parent among the candidate parents set $CP_{np}$; this selection is achieved according to the updated probabilistic routing table, defined by the update of the initial probabilistic routing table. For the nodes in $CP_{np}$ at the time when $n_{h}$ (parent node of $n_{np})\;$loses $\ell_{e}\;$of its energy, the update is calculated by Equation (12) as following:

$$\tilde{h}\left(n_{np,j}\right)=\frac{{\left(1-\overset{ˇ}{h}\left(n_{np,j}\right)\right)}^{\alpha }}{\sum_{l=1}^{C_{np}}{\left(1-\overset{ˇ}{h}\left(n_{np,l}\right)\right)}^{\alpha }}\;,\;\;$$

$${\tilde{d}}_{np,j}=\frac{{\left(1-{\overset{ˇ}{d}}_{np,j}\right)}^{\beta }}{\sum_{l=1}^{C_{np}}{\left(1-{\overset{ˇ}{d}}_{np,l}\right)}^{\beta }}\;\;and$$

$${\tilde{E}}_{np,j}=\frac{{\bar{E}}_{np,j}{}^{\;\gamma }}{\sum_{l=1}^{C_{np}}{\bar{E}}_{np,l}{}^{\;\gamma }},\;\forall n_{j}\in CP_{np},\;\left|CP_{np}\right|=C_{np}.$$

$${\tilde{\varphi }}_{\left(np,j\right)}=\frac{{\overset{ˇ}{\varphi }}_{\left(np,j\right)}}{\sum_{n_{j}⋃CP_{np}}{\overset{ˇ}{\varphi }}_{\left(np,j\right)}}=\frac{{\left(1-\overset{ˇ}{h}\left(n_{np,j}\right)\right)}^{\alpha }{\left(1-{\overset{ˇ}{d}}_{np,j}\right)}^{\beta }{\bar{E}}_{np,j}{}^{\;\gamma }}{\sum_{l=1}^{C_{np}}{\left(1-\overset{ˇ}{h}\left(n_{np,l}\right)\right)}^{\alpha }{\left(1-{\overset{ˇ}{d}}_{np,j}\right)}^{\beta }{\bar{E}}_{np,l}{}^{\;\gamma }},$$

**Remark 4.**  *Hops-count distribution and the residual energy distribution must be updated, because they may change after the packets transmission process, while the transmission distance distribution has to be updated not due to the fact that it changes by time (the nodes are statics), but due to the fact that the neighboring nodes set of the node needing new-parent* $n_{np\;}$ *changes, which requests a transmission distance distribution update.*

#### 4.3.3. Updating the Hops-Count for the Sub-Tree ($n_{np})$ Nodes

In each time any node $n_{np}\;$changes its parent, and the hop-count of its new-parent is different from the hop-count of its old-parent, the hop-count of the all nodes belonging to the Sub-tree ($n_{np}$) has to be updated according to Equation (18), thus the update of the hops-count distribution and the update of the probabilistic routing table by Equation (12) are completed.

After linking the node needing new-parent $n_{np}$ to its new-parent$\;n_{p}$, if$\;h\left(n_{p}\right)\neq h\left(n_{h}\right),$ then for each child node and its parent node in Sub-tree ($n_{np}$), the hop-count should be updated by the following Equation (18).

(18) $$h\left(n_{np}\right)=h\left(n_{p}\right)+1\;and\;h\left(child\;node\right)=h\left(parent\;node\right)+1$$

## 5. Analysis

### 5.1. Expected Data Forwarding Probability

Each node $n_{i}\;$has to forward its sensed and collected data according to an expected data forwarding probability $FP_{i}$. Let $\partial_{i}=\sum_{l=1}^{m_{i}}{\left(1-\overset{ˇ}{h}\left(n_{i,l}\right)\right)}^{\alpha }\sum_{l=1}^{m_{i}}{\left(1-{\overset{ˇ}{d}}_{i,l}\right)}^{\beta }\;\sum_{l=1}^{m_{i}}{\bar{E}}_{i,l}{}^{\;\gamma }\;$and $\rho_{i}=\sum_{l=1}^{m_{i}}{\left(1-\overset{ˇ}{h}\left(n_{i,l}\right)\right)}^{\alpha }{\left(1-{\overset{ˇ}{d}}_{i,l}\right)}^{\beta }\;{\bar{E}}_{i,l}{}^{\;\gamma }$ and${\;\omega }_{i,j}={\left(1-\overset{ˇ}{h}\left(n_{i,j}\right)\right)}^{\alpha }{\left(1-{\overset{ˇ}{d}}_{i,j}\right)}^{\beta }{\bar{E}}_{i,l}{}^{\;\gamma }$.

By replacing in Equations (11) and (12), we get: ${\overset{ˇ}{\varphi }}_{i,j}=\frac{\omega_{i,j}}{\partial_{i}}$ and ${\tilde{\varphi }}_{i,j}=$ $\frac{\omega_{i,j}}{\rho_{i}}$.

Hence, the expected data forwarding probability for the node $n_{i}$ is given by Equation (19).

(19) $$FP_{i}=\overset{m_{i}}{\underset{j=1}{\sum }}{\overset{ˇ}{\varphi }}_{i,j}{\tilde{\varphi }}_{i,j}=\overset{m_{i}}{\underset{j=1}{\sum }}\frac{\omega_{i,j}}{\partial_{i}}(\frac{\omega_{i,j}}{\rho_{i}})=\frac{1}{\partial_{i}\rho_{i}}\overset{m_{i}}{\underset{j=1}{\sum }}\omega_{i,j}{}^{2}.$$

### 5.2. Variance

Since the residual energy varies after each energy consumption when sending a packet, and since the hop-count distribution varies after the update of the tree, the expected data forwarding probability given by Equation (19) varies by the variance $\sigma_{i}^{2}$, given by Equation (20) for the source node $n_{i}.$

(20) $$\sigma_{i}^{2}=\overset{m_{i}}{\underset{j=1}{\sum }}{\tilde{\varphi }}_{i,j}{\left(\;{\overset{ˇ}{\varphi }}_{i,j}-EX_{i}\right)}^{2}=\overset{m_{i}}{\underset{j=1}{\sum }}\;\frac{\omega_{i,j}}{\rho_{i}}\;{\left[\frac{\omega_{i,j}}{\partial_{i}}-\frac{1}{\partial_{i}\rho_{i}}\overset{m_{i}}{\underset{j=1}{\sum }}\omega_{i,j}{}^{2}\right]}^{2}.$$

### 5.3. Path Expected Energy Cost

Let $E_{p_{n_{s}}^{k}}\;\mathrm{be}\;$the expected energy cost of the path $p_{n_{s}}^{k}$ transmitting a packet of size k from the source node $n_{s}\;$to the sink node $n_{b}$ via a tree structure, such that $p_{n_{s}}^{k}=\left\{n_{s},n_{v},n_{v},n_{w},\ldots ,n_{l},n_{b}\right\}$. $E_{p_{n_{s}}^{k}}\;$is given Equation (21) and defined as the sum of the energy cost of all path nodes. From Equations (1) and (2), we get:

(21) $$E_{p_{n_{s}}^{k}}=\underset{\forall \langle n_{i},n_{j}\rangle \in p^{n_{s}}}{\sum }E_{Tx}\left(\langle n_{i},n_{j}\rangle ,k\right)+\underset{\forall \langle n_{i},n_{j}\rangle \in p^{n_{s}}}{\sum }\;E_{Rx}\left(n_{j},k\right)$$

## 6. Simulation Results

To prove the performance of the proposed PDTR protocol, a designed toolkit using C# and WPF was developed. The simulation had the following aims and results.

### 6.1. Impact of Loss of Energy $\ell_{e}$ Parameter

#### 6.1.1. On the Energy Consumption

Ensuring longer network lifetime when minimizing the energy consumption at the same time is the goal of the best WSN routing protocol. In this paper, we proposed a new tree-based strategy that has an update phase each time any node loses $\ell_{e}$ of its energy.

For this, we considered three scenarios; in each scenario, each source node sends one packet every 0.1 s, for the parameters defined in Table 1 and their values given by Table 2. 

**Figure 3:**
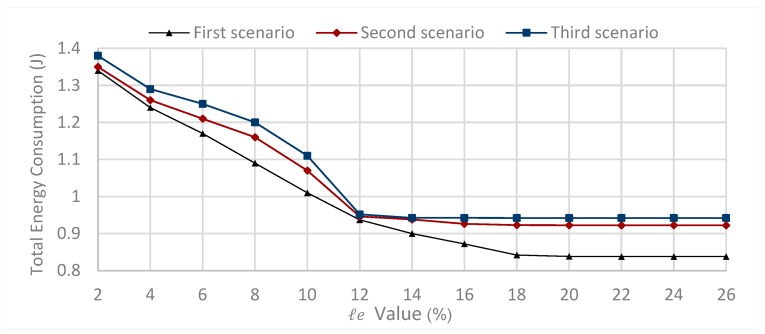
Impact of $\ell_{e}$ parameter on the total energy consumption varying number of nodes for the first scenario, the second scenario and the third scenario.

The communication range R=80 m, $\mathrm{while}\;\ell_{e}=\left\{2\%,4\%,6\%,8\%,10\%,12\%,14\%,16\%,18\%,20\%,22\%,24\%,26\%\right\}$. In the first scenario, the number of nodes is fixed, such that N=100. Figure 3, first scenario shows the simulation results of sending, in each t = 0.1 s, one packet from one random node towards the sink for a period of 300 s. The results show that to minimize the network energy consumption, the value of $\ell_{e}\;$parameter should belong to $\ell_{e}=\left[20\%,26\%\right].$ (see Figure 3, first scenario).

In the second scenario, the number of nodes is fixed such that N=120. Figure 3, second scenario shows the simulation results of sending, in each t = 0.1s, one packet from one random node towards the sink for a period of 300 s. The results show that to minimize the network energy consumption, the value of $\ell_{e}\;$parameter should belong to [16%,26%] (see Figure 3, second scenario). 

In the third scenario, the number of nodes is fixed such that N=140. Figure 3, third scenario shows the simulation results of sending, in each t = 0.1 s, one packet from one random node towards the sink for a period of 300 s. The results show that to minimize the network energy consumption, the value of $\ell_{e}\;$parameter should belong to [14%,26%] (see Figure 3, third scenario).

From the above three scenarios, we can conclude that minimize the total energy consumption, the selection of $\ell_{e}\;\mathrm{value}\;$depends on the number of nodes. For the network size N=100, The value of $\ell_{e}\;$parameter should belong to $\ell_{e}=\left[20\%,26\%\right]$ for the network size N=120, The value of $\ell_{e}\;$parameter should belong to $\ell_{e}=\left[16\%,26\%\right]$ for the network size N=140, The value of $\ell_{e}\;$parameter should belong to $\ell_{e}=\left[14\%,26\%\right]$ for the parameter values given by Table 2, and the communication range R=80 m.

#### 6.1.2. On the Network Lifetime

Prolonging the network lifetime can be reached by saving the nodes’ residual energy. To maximize the network lifetime, we should study the impact of the loss of the energy parameter.

**Figure 4:**
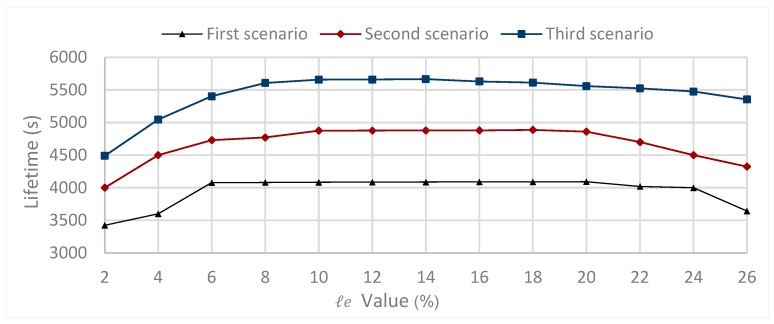
Impact of $\ell_{e}$ parameter on the network lifetime varying number of nodes for the first scenario, the second scenario and the third scenario.

For this, we consider three scenarios; in each scenario, each source node sends one packet every 0.1 s for the parameter values given by Table 2. The communication rangeR=80 m, $\mathrm{while}\;\ell_{e}=\left\{2\%,4\%,6\%,8\%,10\%,12\%,14\%,16\%,18\%,20\%,22\%,24\%,26\%\right\}$. In the first scenario, the number of nodes is fixed such that N=100. In Figure 4, the first scenario shows the simulation results after the round when the first node is declared dead. The results show that, to increase the network lifetime, the value of $\ell_{e}\;$parameter should be set such that $\ell_{e}=20\%$.

In the second scenario, the number of nodes is fixed such that N=120. In Figure 4, the second scenario shows the simulation results after the round when the first node is declared dead. The results show that, to increase the network lifetime, the value of $\ell_{e}\;$parameter should be set such that $\ell_{e}=18\%$. 

In the third scenario, the number of nodes is fixed such that N=140. Figure 4, third scenario shows the simulation results after the round when the first node is declared dead. The results show that, to increase the network lifetime, the value of $\ell_{e}\;$parameter should be set such that $\ell_{e}=14\%$.

From the above three scenarios, we can conclude that there is a decreasing relationship between the network size (N) and the optimal value of the loss energy parameter $\ell_{e}\;$that maximizes the network lifetime. It is concluded that the maximum lifetime is reached when $\ell_{e}=20\%$ for the network size N=100 , the maximum lifetime is reached when $\ell_{e}=18\%$ for the network size N=120, and the maximum lifetime is reached when $\ell_{e}=14\%$ for the network size N=140 for the parameters defined in Table 1 and their values given by Table 2. The communication rangeR=80 m.

### 6.2. Control Parameters Upper Bound

The user has to decide the values of the control parameters, depending on the requirement of the applications. The role of each parameter is to determine the impact of the corresponding distribution on the other distributions. The values of the control parameters should be selected in order to keep the influence of the three distributions. After intensive experimental work, it was concluded that to maintain this objective, the upper bound of control-parameters denoted by $U_{b},\;$has a strong relation with the network density deployment; this relation is defined by Equation (26). In the homogeneous network, sensors are deployed at the interest network area Ω, and they have the same communication radius, denoted by δ. To calculate the density of the deployment of these N sensors on the given network, we consider two parts. First, the area of each sensor in the network is denoted by $S_{n_{i}}$ (it is the same for all sensors, since sensors are homogenous), and is defined by Equation (22). Second, the average of sensors’ neighboring nodes in the network, denoted by $\bar{M}$ and defined by Equation (23). The density of sensor node deployment in the network is denoted by D, and is defined by Equation (25), and the sum of the area of all sensors is denoted by $S_{N}$, and is defined by Equation (24), par the interest network area Ω.

(22) $$\forall n_{i}\in \mathbb{N},\;\;S_{n_{i}}=\pi \delta^{2}\;$$

(23) $$\bar{M}=\frac{\sum_{0}^{n}m_{i}}{n},\;such\;that:\;\left|N_{i}\right|=m_{i}$$

(24) $$S_{N}≅S_{n_{i}}\left(\frac{n+\bar{M}}{2}\right)\;≅\pi \delta^{2}\left(\frac{n+\frac{\sum_{0}^{n}m_{i}}{n}}{2}\right),$$

(25) $$D≅\frac{S_{n_{i}}\left(\frac{n+\bar{M_{i}}}{2}\right)\;}{\Omega }=\frac{S_{n_{i}}\left(n+\bar{M_{i}}\right)\;}{2\Omega },$$

(26) $$U_{b}=2D.\;(\mathrm{Experimental}\;\mathrm{view})$$

### 6.3. Importance of Implementing the Control-Parameters Sets on PDTR 

#### 6.3.1. On the Delay

Achieving the low delay when sending and receiving data can be reached by minimizing the number of hops in the routing protocol. Hence, the control parameter of the hop-count distribution α should have greater value than the two other control parameters. 

**Figure 5:**
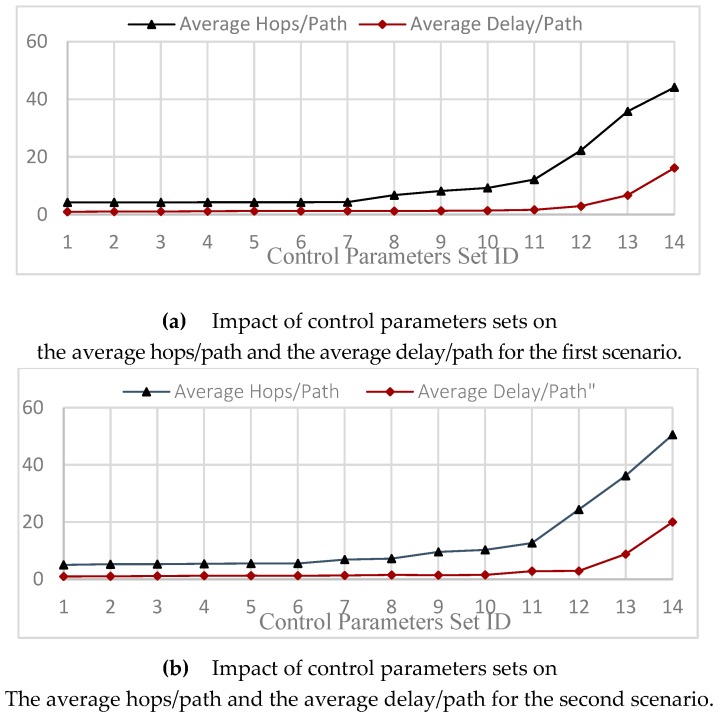
Impact of control parameters sets on the average hops/path and the average delay/path.

In the first scenario, 14 parameters set are selected such that the value of α=2, while γ=β={0,0.2,0.4,0.6,0.8,1,1.2,1.4,1.6,1.8,2,2.2,2.4,2.6}. Each source node 1 < s < 100, sends one packet each 0.1 s, and the simulation time is 300 s, for the parameter values given by Table 2, such that the communication range is R = 50. The results show that the minimum number of hops in the routing protocol is reached when γ=β=0, (see Figure 5a).

In the second scenario, 14 parameters set are selected such that the value of α=3, while γ=β={0,0.2,0.4,0.6,0.8,1,1.2,1.4,1.6,1.8,2,2.2,2.4,2.6}. Each source node 1 < s < 100, sends 1packet every 0.1 s, and the simulation time is 300 s, for the parameter values defined and given by Table 1 and Table 2, such that the communication range is R = 50. The results show that the minimum number of hops in the routing protocol is reached when γ=β=0, (see Figure 5b).

From the above two scenarios, we can conclude that; first, the minimum number of hops in the proposed PDTR protocol is reached when γ=β=0 . Second, the greater hop-count control parameter, the lower the number of hops needed in the PDTR routing protocol. 

This conclusion is maintained under the condition: $\alpha +\gamma +\beta \leq U_{b}\;$(Experimental view).

#### 6.3.2. On the Energy Consumption

Minimizing both the transmission distance and the number of hops leads to minimizing energy consumption. To achieve this goal, the control parameters of both the transmission distance and the hop-count have to be greater than the control parameter of the residual energy distribution. For this, we consider three scenarios. 

**Figure 6:**
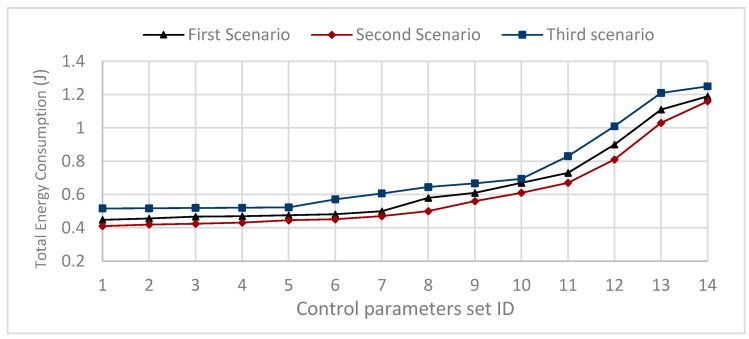
Impact of control parameters sets on total energy consumption for first scenario, second scenario and third scenario.

The first scenario selected 14 parameters set such that the value of α=3, β=1, while γ={0,0.2,0.4,0.6,0.8,1,1.2,1.4,1.6,1.8,2,2.2,2.4,2.6}. Each source node sends one packet every 0.1 s, and the simulation time is 100 s, for the parameter values defined in Table 1 and given by Table 2, and the communication radius R = 25 m. The results show that to achieve the minimum energy consumption, the control parameter of the residual energy should be γ = 0, (see Figure 6, first scenario).

The second scenario selected 14 parameters set such that the value of α=3, β=2, while γ={0,0.2,0.4,0.6,0.8,1,1.2,1.4,1.6,1.8,2,2.2,2.4,2.6}. Each source node sends one packet every 0.1 s, while the simulation time is 100 s, for the parameter values given by Table 2, and the communication range R = 50 m. The results show that to achieve the minimum energy consumption, the control parameter of the residual energy should be γ = 0, (see Figure 6, second scenario).

The third scenario selected 14 parameters set such that the value of α=3, β=3, while  γ={0,0.2,0.4,0.6,0.8,1,1.2,1.4,1.6,1.8,2,2.2,2.4,2.6}. Each source node sends one packet every 0.1 s, and the simulation time is 100 s, for the parameter values given by Table 2, and the communication range R = 50 m. The results show that to achieve the minimum energy consumption, the control parameter of the residual energy should be γ = 0, (see Figure 6, third scenario).

From the above three scenarios, we can conclude that; first, the minimum energy consumption in the proposed PDTR protocol is reached when γ=0. Second, for better results in term of energy consumption, the control parameter of the transmission distance should be set such that: $\beta >\frac{\alpha }{2}.\;$Third, for better results in term of energy consumption, the control parameter of the transmission distance should be less than the control parameter of the hops-count β<α.To minimize the energy consumption in the PDTR routing protocol, the values of the control parameters should be set such that$\;\gamma =0,\;\alpha >\beta >\frac{\alpha }{2}.$This conclusion is maintained under the condition: $\alpha +\gamma +\beta \leq U_{b}$ (Experimental view).

#### 6.3.3. On the Network Lifetime

Prolonging the network lifetime can be achieved by selecting the nodes with higher residual energy. To maximize the network lifetime, we should maximize the value of the residual energy control parameter γ. 

**Figure 7:**
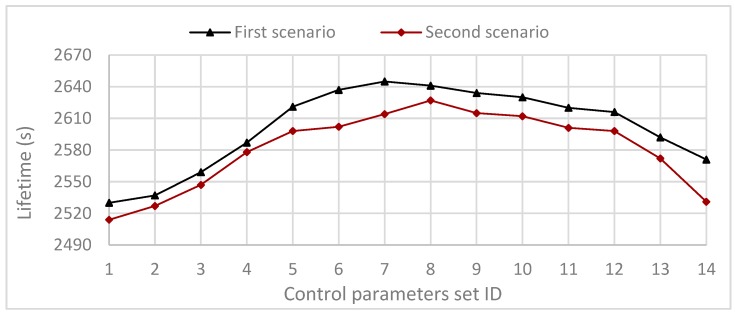
Impact of control parameters sets on the network lifetime for first scenario and second scenario.

For this, we consider two scenarios. The first scenario selected 11 parameters set such that the value of α=3, β=2, while γ={0,0.2,0.4,0.6,0.8,1,1.2,1.4,1.6,1.8,2}. Each source node sends one packet every 0.1 s for the parameter values given by Table 2, the communication range is fixed R=50 m, and the number of nodes is fixed N = 100. Figure 7, first scenario shows the simulation results after the round when the first node is declared dead. It shows that the maximum lifetime is reached when γ=1.2. 

The second scenario selected 11 parameters set such that the value of α=3, β=2.6, while γ={0,0.2,0.4,0.6,0.8,1,1.2,1.4,1.6,1.8,2}. Each source node sends one packet every 0.1 s for the parameter values given by Table 2, the communication range is fixed R=50 m, and the number of nodes is fixed N = 100. Figure 7, second scenario shows the simulation results after the round when the first node is declared dead. It shows that the maximum lifetime is reached when γ=1.4.

From the above two scenarios, we can conclude that the maximum network lifetime in the proposed PDTR protocol is reached when: $\frac{3\beta }{4}>\gamma >\frac{\beta }{2};$ for $\alpha +\gamma +\beta \leq U_{b}$, and $\frac{3\alpha }{4}>\beta >\frac{\alpha }{2}$.

### 6.4. Performance Comparison of the Proposed PDTR to the State-of-the-Art Routing Protocols

To prove the performance of our proposed approach PDTR, we compare it to the following protocols.

**Figure 8:**
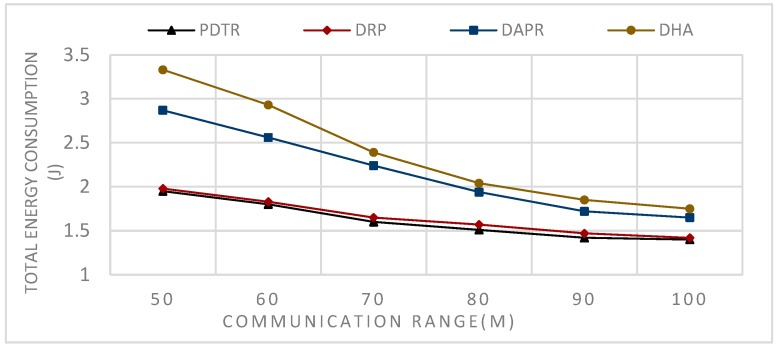
Impact of varying communication range on total energy consumption.

**Figure 9:**
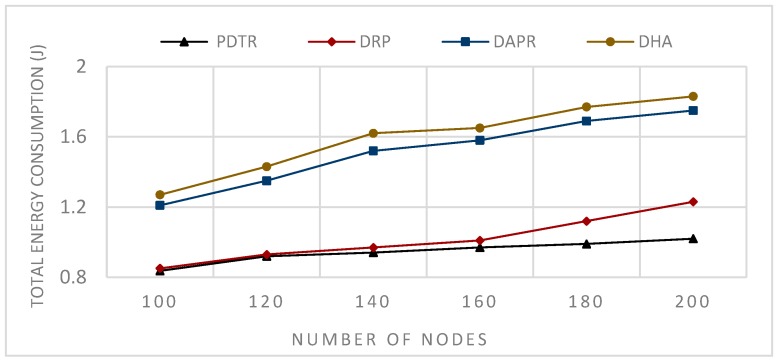
Impact of varying number of nodes on total energy consumption.

In Deterministic Routing Protocols (DRP), such as Tree Routing (TR) and Enhanced Tree Routing (ETR) the paths are predefined, and each node has a fixed forwarder node. Thus, the protocol does not need more energy to be updated, unlike the case of probabilistic protocols; consequently, the DRP consumes less energy and provides better performance in terms of energy consumption in comparison to the probabilistic protocols, as we can see in Figure 8 and Figure 9. 

**Figure 10:**
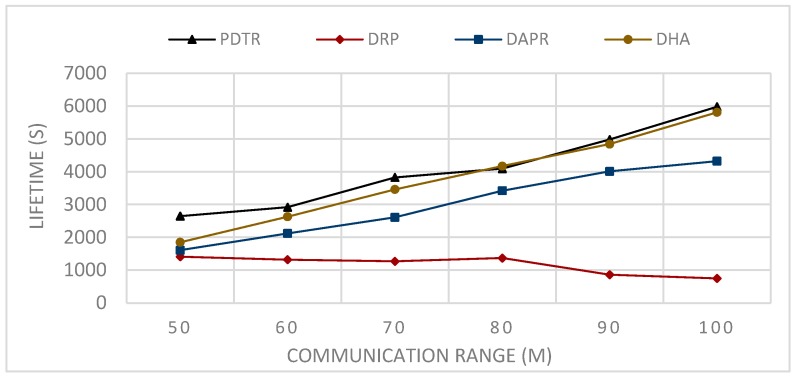
Impact of varying the communication range on the network lifetime.

**Figure 11:**
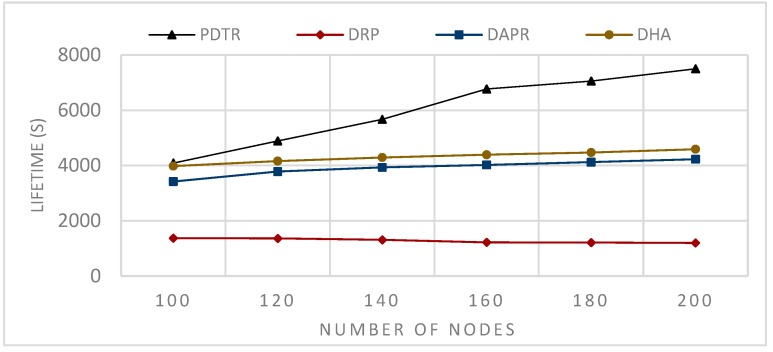
Impact of varying the number of nodes on the network lifetime.

Moreover, DPR, with its unicast routing, is the worst in terms of energy balancing and lifetime, since some nodes will be more loaded than others (see Figure 10 and Figure 11).

**Figure 12:**
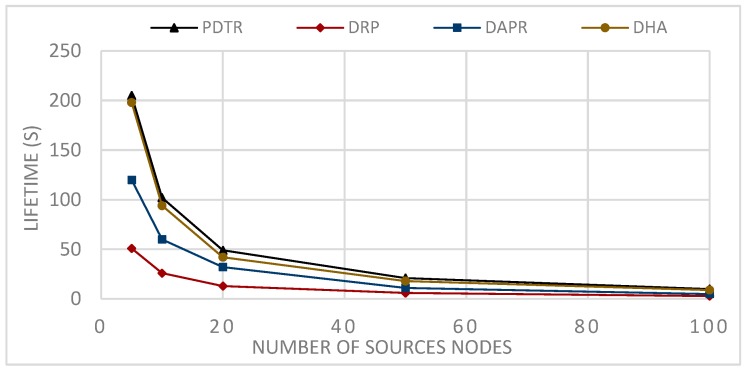
Impact of varying the number of sources nodes on the network lifetime.

**Figure 13:**
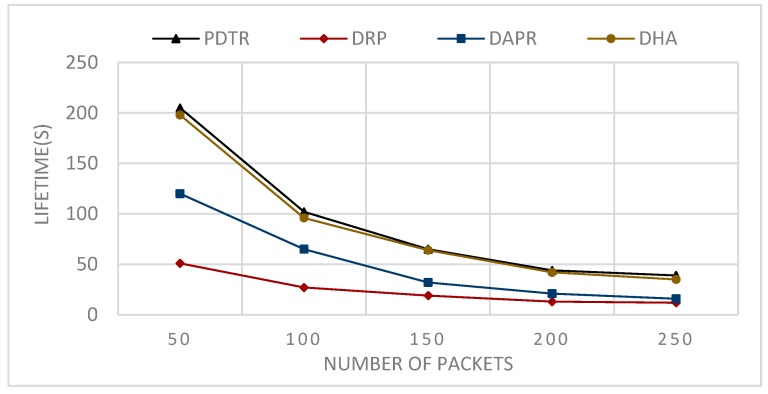
Impact of varying the number of packets on the network lifetime.

However, the fact that each node has only one fixed forwarder means that each time the sender node sends data to its parent if its parent is in sleep mode, the sender node has to wait, which means an increase in the waiting time delay, thus an increase in the sending time delay, and a decrease in the number of generated packets in comparison to probabilistic protocols, (see Figure 12 and Figure 13). Our proposed approach with the parent probabilistic selection decreases the delay in both the waiting time and sending time. The PDTR at the update phase offers for the node needing a parent a set of candidate parents with different probabilities, and the node has to choose the suitable awake parent through a tree structure. PDTR also improves on DRP in terms of energy efficiency since it builds the tree based on three probability distributions (hops count, transmission distance and residual energy). Since the node’s initial energy is the same in our case, we do not consider this criterion. Based on these criteria, PDTR starts generating packets through best-selected paths, and over time the network loading will be high for some nodes (node loses $\ell_{e}$ =20% of its energy for this scenario (Network size = 100)), and the PDTR starts the probabilistic update; thus it achieves better performance than DRPs in term of energy balancing and energy efficiency.

In Probabilistic Routing Protocols (PRP), such as Distributed Adaptive Probabilistic Routing (DAPR) and Distributed Heuristic Algorithm (DHA)), unlike the DRP, the sender in PRP has a set of candidate parent nodes, thus transmitting packets via different paths and distributing the network load and the energy consumption among nodes; consequently, PRPs improved on DRPs in terms of network balancing and lifetime, (see Figure 10 and Figure 11). Moreover, the fact that the nodes have more candidate nodes when sending the packets means that the problem of the forwarder being in sleep mode does not affect the transmission, because the node has to choose another appropriate awake forwarder, thus decreasing both the waiting time and sending time in comparison to the DRPs, and increasing the number of generated packets (see Figure 12 and Figure 13). However, in term of energy consumption, PRPs need more energy than DRPs, first because the packets may travel via longer paths, and second due to the updating, these protocols are built with the probabilities that need to be adjusted according to the transmission process; for these reasons, PRPs need more energy consumption than DRPs (see Figure 8 and Figure 9). PDTR improved on PRPs in term of energy consumption by the fact that it processes via a tree structure based on 3PD probability distributions (Hops distribution, transmission distance distribution, and residual energy distribution), and packets traveling via the tree structure built from optimal paths need less energy consumption than traveling via anycast structure. Moreover, PDTR has to be updated each time the node meets the high network loading, in order to save the energy and prolong the lifetime; thus, the PDTR increases the lifetime of DRP and PRP cited above (see Figure 10, Figure 11, Figure 12 and Figure 13). On the other hand, the updating of PDTR does not need much energy, unlike in PRPs, due to the fact that the update of PDTR happens only when any node loses 20% of its energy. Hence, better performance in terms of energy consumption (see Figure 8 and Figure 9).

Figure 8 shows the simulation results of sending, in each t = 0.1 s, one packet from one random node towards the sink for a period of 480 s; the number of nodes is assumed fixed N=100, while the communication range R varies, such that: R = {50,60,70,80,90,and 100 m}.

Figure 9 shows the simulation results of sending, in each t = 0.1 s, one packet from one random node towards the sink for a period of 300 s; the communication range is assumed fixed R=80 m, while the number of nodes N varies, N={100,120,140,160,180,200}.

Figure 10 shows the simulation results after the round when the first node is declared dead of sending, in each t = 0.1 s, one packet from one random node towards the sink; the number of nodes N is assumed to be fixed N=100, while the communication range R varies, R={50,60,70,80,90,and 100 m}.

Figure 11 shows the simulation results after the round when the first node is declared dead of sending, in each t = 0.1 s, one packet from one random source node towards the sink, the communication range is assumed to be fixed R=80 m, while the number of nodes N varies, N={100,120,140,160,180,200}.

Figure 12 shows the simulation results after the round when the first node is declared dead of sending in each round 50 packets (1packet/1 s) from each source node among the randomly selected source nodes SN, such that: SN={5,10,20,50,100}. 

Figure 13 shows the simulation results after the round when the first node is declared dead of sending in each round (second) an NP packet (1packet/1 s) from each source node among five randomly selected source nodes, such that NP varies from 50 to 250. 

The simulation results prove that our proposed algorithm PDTR provides better performance than DRP, DAPR, and DHA in terms of energy consumption and lifetime.

From Figure 8, we can conclude that the relation between the communication range and the energy consumption is a decreasing relation—larger communication range leads to less energy consumption. This can be explained by the fact that extending the communication range will reduce the number of hops needed to transmit packets towards the sink. From Figure 9, we can conclude that the relation between the number of nodes and the energy consumption is an increasing relation. This can be explained by the fact that a larger network size means a longer paths length, thus higher path expected energy cost. From Figure 10, we can conclude that there is an increasing relation between the lifetime and the communication range; this can be concluded from the conclusion obtained from Figure 8. Larger communication range leads to lower energy consumption, and hence longer lifetime. From Figure 11, we can conclude that there is an increasing relationship between the lifetime and the network size. Despite the conclusion obtained from Figure 8, the larger network size leads to higher energy consumption. PDTR prolongs the lifetime when increasing the size of the network size, due to the fact that more nodes mean more possible forwarder candidates in a probabilistic strategy, thus providing a longer lifetime. From Figure 12, we can conclude that there is a decreasing relationship between the lifetime and the number of source nodes. This can be explained by the fact that more source nodes means more generated packets, and more generated packets need more energy consumption, hence leading to a shorter lifetime. From Figure 13, we can conclude that there is a decreasing relationship between the lifetime and the number of generated packets; as explained above, more generated packets need more energy consumption, thus leading to shorter lifetime.

## 7. Conclusions

This paper presents a Probabilistic and Deterministic Tree Routing Protocol called PDTR. It aims to root the packet toward the sink via optimal tree paths and save the nodes’ energy for as long as possible. PDTR simulation results show a longer network lifetime, and less energy consumption, in comparison to the previously proposed protocols. That is to say that PDTR is a suggested routing protocol to prolong the network lifetime under the condition of sensor homogeneity.

PDTR improves the deterministic tree strategy due to the fact that it builds the tree according to three probability distributions (hops-count, transmission distance, and residual energy). Hence the selection of optimal paths and the saving of energy. Moreover, PDTR improves the probabilistic tree strategy. First, this is due to the fact that it forwards data based on the product of three probability distributions controlled by parameters, two of them need to be updated by time (residual energy and hops-count); this update performs the use of paths when transmitting data, in comparison to the protocols based on predefined probability distribution. Second, it does not need much energy to be updated, in comparison to the other probabilistic protocols, because the update of PDTR happens only when any node loses $\ell_{e}$ of its energy, hence a better performance in terms of energy consumption is obtained.

Our work is based on hop-count distribution and residual energy, which sometimes need to be updated in the tree due to the change in sub-trees position and nodes’ residual energies. This update needs some energy. Hence, our future work involves examining this fairness issue in order to enhance the PDTR and save the used energy to prolong the WSN lifetime. 

## Figures and Tables

**Table 1:** Notations.

Notation	Description
ℕ	The set of sensor nodes in the network; $\mathbb{N}=\left\{n_{0},n_{1},n_{2},\ldots \right\};$
$\;{\mathbb{N}}_{i}$	The neighboring nodes of the node $n_{i};$ $m_{i}=\left|{\mathbb{N}}_{i}\right|$.
$n_{h}$	Node needing help, is defined by the node that loses $\ell_{e}\;$of its initial energy.
$n_{np}$	Node needing new-parent, is defined as the removed child node from $\;n_{h}\;$children, when updating the tree.
$d_{i,j}$	The Euclidian distance between: $n_{i},n_{j}$.
$h\left(n_{i}\right)$	The hop-count of the node $n_{i}$ , is the minimum number of hops to reach the sink.
$E_{i}$	The residual energy of the node $n_{i}.$
δ	The communication radius.
α	The control parameter of the impact of hop-count distribution on the routing protocol.
β	The control parameter of the impact of transmission distance distribution on the routing protocol.
γ	The control parameter of the impact of the residual energy distribution on the routing protocol.
${\tilde{\varphi }}_{i,j}{}^{*}$	The initial probability of the link $n_{i},n_{j}$, is defined by Equation (14).
${\tilde{\varphi }}_{i,j}$	The probability of the link $n_{i},n_{j}$, is defined by Equation (12).
$p_{n_{s}}^{k}$	The path transmitting a packet of size k, from the source node $n_{s}\;$to the sink node $n_{b}$, is defined by Equation (15).
$Sbt_{N}^{n_{q}}$	The nodes of the Sub-tree ($n_{q}$), sub-tree rooted at the node $n_{q}$, is defined by Equation (16).
$CP_{np}$	The set of candidate parents of the node need new-parent $n_{np};\left|CP_{np}\right|=C_{np}$, is defined by Equation (17).
$FP_{i}$	The expected data forwarding probability $FP_{i}$ for the node $n_{i},$ it is defined by Equation (19).
$\sigma_{i}^{2}$	The variance of the Expected data forwarding, it is given by Equation (20) for the source node $n_{i}.$
$E_{p_{n_{s}}^{k}}$	The expected energy cost of the path $p_{n_{s}}^{k},\;$transmitting a packet of size k, from the source node $n_{s}\;$to the sink node $n_{b},$ is defined by Equation (21).
$\ell_{e}$	The loss of energy.
$\;\;\;S_{n_{i}}$	The area of the sensor $n_{i}$, is defined by Equation (22).
$\;\;S_{N}$	The total area of the sensors in the network N, is defined by Equation (24).
$\bar{M}$	The average of all neighboring nodes in N, is defined by Equation (23).
D	The network density, is defined by Equation (25)

**Table 2:** Parameters values.

Parameter	Value
Network Topology	Tree Topology
Network Size	100 × 100 m^2^
Number of Nodes	100 (Static nodes)
Sink	1 Static sink (fixed at the center)
Communication Radius	25–50 m
Initial Energy for all nodes	0.5 Joule/ except when other assumption.
Sink Initial Energy	500 Joule
Radio Propagation Model	First Radio model
MAC	BOX-MAC
Packet Size	1024 bits
α	3 by assumption/except if other assumption.
β	1.8 by assumption/except if other assumption.
γ	1 by assumption/ except if other assumption.
